# Abstract of Report and Clinics of the Southern Dental Association

**Published:** 1898-01

**Authors:** 


### Abstract of Report and Clinics of the Southern Dental Association.

Read before Southern Dental Association, Old Point Comfort, August, 1897.

Dr. R. Ottolingui demonstrated the value of Williams' platina gold-foil in restoring incisive edges of badly abraded teeth, using the Custer gold-annealer, and packing with the Bonwill engine mallet.

Dr. C. L. Alexander demonstrated the mechanism of a new method of crowning a tooth, both the porcelain crown and the root of the natural tooth being provided with a cap and post. The parts when placed in position are held together with wax until removed and soldered. By this method the porcelain crown may be placed at any angle upon the tooth to be crowned.

Dr. L. E. Custer demonstrated his improved electric oven. The cause of the burn-out, which occurred in his first ovens has been discovered and corrected, so that a burn-out can not now occur in the legitimate use of the improved oven.

Dr. V. H. Jackson demonstrated his crib-anchorage in regulating cases.

Dr. W. E. Walker demonstrated his facial clinometer for recording the condylo-occlusal angle of the patient by which to set the Walker physiological articulator, in order to secure a "balancing articulation."

Dr. W. J. Brady demonstrated the use of the angle-regulating appliances on twelve cases selected from his practice, with models showing the results attained.

Dr. F. F. Fletcher demonstrated the advantages of a narrow saddle in bridge-work; also, demonstrated that the piers in bridge-work should be parallel, making them so by regulating when necessary, the bridge acting as a retaining appliance.

Dr. V. De Trey demonstrated the use of solid gold, using special instruments; no undercuts or retaining points, the grooves left by the cross-cut burs being sufficient to retain the filling.

Dr R. Eugene Payne gave a clinic in implantation of the superior right central incisor, sterilized gum and mouth, with borolyptol followed by alcohol at point of operation; cocainize the gum and cut double flap; with Ottolingui bone drill cut socket full depth, using great care to preserve outer thin plate; used small, slim, mature root, dense, hard and round, with perfect pericemental membrane, sterilized in 1-5000 bichloride over night; cut off worn natural crown and fitted a Davis crown, shortening the root without cutting apical end of root; filled root with paraffin and salol; washed out socket with borolyptol, and just before final insertion of tooth placed a few crystals resorcin on root to make sure no infection develop in socket before nature protects the wound; ligated with "00" silk twist; instruments sterilized with aqua ammonia; tooth in 1-3000 bichloride; hands with alcohol.

Dr. C. M. Carr demonstrated his method of "anchored adjustable dentures." So anchored as to prevent displacement, but adjustable and removable by patient at will.

